# The impact of occupational and personal factors on musculoskeletal pain - a cohort study of female nurses, sonographers and teachers

**DOI:** 10.1186/s12891-020-03640-4

**Published:** 2020-09-18

**Authors:** Inger Arvidsson, Jenny Gremark Simonsen, Agneta Lindegård-Andersson, Jonas Björk, Catarina Nordander

**Affiliations:** 1 Division of Occupational and Environmental Medicine, Lund University, SE-221 85 Lund, Sweden; 2 Institute of Stress Medicine, Carl Skottsbergs gata 22B, SE-413 19 Gothenburg, Sweden

**Keywords:** Longitudinal study, Musculoskeletal disorders, Multivariable model, Multisite pain, Regional pain

## Abstract

**Background:**

Musculoskeletal pain is common in the general population and constitutes a major public health problem*.* A large proportion of these conditions may be work related. The aim of this study was to explore the relative importance of physical, psychosocial and personal factors, in number of pain sites and in five specific pain sites, among women in common professions with a broad variety of occupational exposures.

**Methods:**

A cohort of 1115 women responded to a questionnaire on ergonomic, psychosocial, personal and life-style factors, and the outcome measure of musculoskeletal pain (based on frequency and intensity of complaints at nine anatomical sites), at baseline and at follow-up. Sum scores of ergonomic and psychosocial factors were created. The importance of exposure at baseline for the number of pain sites at follow-up were estimated using ordinal regression. The importance of exposure at baseline for pain in the neck, shoulders, hands, lower back and feet at follow-up were estimated using multi-exposure Poisson regression models.

**Results:**

High sum scores for ergonomic and psychosocial factors were of importance for a high number of pain sites, although the strongest risk factor was a high number of pain sites already at baseline. On the individual level, there was a large fluctuation in number of pain sites between the two time points. Eighteen percent reported persistent (or recurrent) ≥ four pain sites, while only 11 % did not report any pain at baseline or at follow-up. Among the specific pain sites, a high sum score of ergonomic factors was associated with pain in the neck, hands and feet. A high sum score of psychosocial factors was associated with neck and shoulder pain. The strongest risk factor was, however, pain at that specific anatomical site at baseline. Only a few of the personal and life-style factors were associated with pain.

**Conclusions:**

An overwhelming majority of the women in common occupations were affected by musculoskeletal pain. Both ergonomic and psychosocial factors were predictive of a high number of pain sites and of specific pain sites. These findings indicate the need for preventive measures on the individual, organizational and societal level.

## Background

Musculoskeletal pain is common and thus constitute a major public health problem [1]*.* In several scientific studies, the authors claim that some of these symptoms/disorders among individuals may be related to exposures that are present at work [2–6]. Increased risk for musculoskeletal pain has been demonstrated for both physical and psychosocial factors*.* A variety of physical exposures, such as constrained postures, high muscular load and forceful exertions, highly repetitive work tasks, lack of time for recovery and poor workstation layout, have been identified as possible physical risk factors [3, 7–9].

Furthermore, previously published scientific studies have found associations between work-related musculoskeletal disorders in the neck/upper extremities and the lower back, and various psychosocial work factors such as high job demands, low job control, job strain, low social support and work stress [5, 10–12].

Various risk factors for musculoskeletal pain have been identified in occupational groups that are highly exposed to physical and/or psychosocial strain. However, it is less clear whether the associations between physical workload and musculoskeletal disorders are similar in occupations where exposures are assumed to be low or intermediate. We have previously carried out a cross-sectional study [13] which served as the baseline of the present cohort study. The aim was to assess and identify risk factors for musculoskeletal pain among females in common occupations, involving many individuals in the society. However, to be able to detect possible associations or causal relationships between physical and psychosocial exposures and pain, there is a need to study contrasting occupational exposures within the cohort. We therefore chose a study population consisting of various healthcare professionals (nurses and sonographers) and teachers in elementary school.

The work tasks of the nurses (i.e. anaesthetic nurses, surgical nurses and assistant nurses) have been reported to include physical exertion when handling patients and equipment, and prolonged twisted and static postures during surgery [14]. The work tasks of the sonographers is, to a high degree, characterized by prolonged sitting in constrained postures [15], while the physical workload among the teachers is varied, and physically relatively light. While time constraints and adverse psychosocial working conditions may be experienced by all five occupational groups, mental strain is particularly high among teachers [13, 16, 17]. Hence, the present study population consists of females in common professions in the public sector, with a broad variety of occupational exposures.

Apart from occupational factors, personal and life-style factors, such as age, obesity, smoking, and lack of physical activity in leisure time [18, 19], may be of importance in the development of musculoskeletal pain. Furthermore, gender also has implications for musculoskeletal pain. There is scientific evidence that women run a higher risk than men [20–22], also when they are performing identical work tasks [21, 23]. As women are particularly affected by musculoskeletal pain, we have chosen to study which risk factors that are present among them in these common female-dominated occupations.

Musculoskeletal pain tends to be intermittent [24] and with varying intensity, but can also develop into a persistent and severe condition. Pain may be limited to a single anatomical site, but may also occur at several sites simultaneously, i.e. multisite pain [25]. The consequences, in terms of consumption of healthcare services, and absenteeism and restrictions at work, have been found to depend on the number of body regions affected [26]. Furthermore, Coggon et al. [27] suggested that the risk factors associated with extensive pain, i.e. pain involving multiple anatomical sites, differs importantly from those associated with limited pain (involving 1–3 anatomical sites). In the present study, we investigated whether this is the case in a cohort of health professionals and teachers. However, there are still inconsistencies in the literature concerning the strength of the evidence for a longitudinal relationship between many of these identified physical and psychosocial risk factors, and musculoskeletal pain [10, 28]*.* Most of the studies performed are cross-sectional, and thus have limited value in studying causal relationships.

In the baseline-study we reported cross-sectional associations between occupational and personal risk factors and the outcomes of pain in the neck, shoulders, hands, lower back and feet [13]. The aim of the present follow-up study was to further explore the relative importance of physical, psychosocial and personal factors, in longitudinal associations with the number of musculoskeletal pain sites, as well as pain at these five specific anatomical sites.

## Methods

### Study design

Questionnaires were used to obtain information from women in five occupational groups, anaesthetic nurses, surgical nurses, assistant nurses, sonographers and teachers, at baseline and at follow-up. Ten items regarding self-assessed occupational exposure and personal factors at baseline were analysed in relation to reported musculoskeletal pain at follow-up. Data were collected on pain at nine anatomical sites; i.e. the neck, shoulders, elbows, hands, upper back, lower back, hips, knees and feet. The importance of potential risk factors was analysed in relation to the number of sites of pain, as well as for pain in five of the anatomical regions, i.e. the neck, shoulders, hands, lower back and feet.

### Study population

All 23 surgical departments in the healthcare regions of southern Sweden were invited to take part in the study at baseline. Invitations were also sent to all 45 departments at hospitals in Sweden where biomedical scientists perform sonography, and to 64 state-run schools from seven counties in southern Sweden. Of these, 22 surgical departments, all 45 sonography departments and 49 schools agreed to participate in the study [13]. The baseline questionnaire was sent to all women in the occupations of interest, which totalled 2078 women at 116 different workplaces. The inclusion criterion was working at least 50% of full-time, during a period of at least 3 months before completing the baseline questionnaire. Of these 2078 women, 1591 participated in the study at baseline [13]. Most of them (91%) worked full-time (≥ 30 h/week) and the mean number of years in their profession (referred to as “seniority”) was 17 (range 0.25–45) years.

Out of the 1591 participants at baseline, 1115 (70%) submitted responses to the follow-up questionnaire. The inclusion criterion was working at least 50% of full-time with the job-specific tasks. The participation rates among the various occupations were:

214 out of the 297 anaesthetic nurses included at baseline (72%); 209/305 surgical nurses (69%); 224/323 assistant nurses (69%); 222/291 sonographers (76%); and 246/375 teachers (66%) (Further information on drop-outs is given in Table 1.)

**Table 1:** Musculoskeletal pain and sum scores for ergonomic and psychosocial factors in the total study population at baseline*;* among the participants at follow-up (*n* = 1115), and among those who dropped out of the study between baseline and follow-up (i.e., “drop-outs”; *N* = 476). The baseline results are given for the participants with partial drop-outs (i.e. with incomplete answers; *n* = 204), who were not included in the adjusted multi-exposure model of multisite pain

		Musculoskeletal pain at baseline						Sum score	Sum score
	All	≥four sites of pain	Neck	Shoulders	Hands	Lower back	Feet	Ergonomic factors	Psychosocial factors
	N	%	%	%	%	%	%	Mean (SD)	Mean (SD)
**Included** at follow-up	**1115**	**26**	**39**	**44**	**24**	**39**	**17**	**2.5 (1.0)**	**1.3 (1.2)**
*Anaesthetic nurses*	214	21	32	40	20	39	17	2.8 (1.0)	1.2 (1.2)
*Surgical nurses*	209	29	39	47	24	42	19	3.3 (0.8)	1.1 (1.1)
*Assistant nurses*	224	36	41	47	37	47	26	2.9 (0.9)	1.3 (1.2)
*Sonographers*	222	24	44	53	25	28	12	2.3 (0.9)	1.0 (1.1)
*Teachers*	246	21	40	34	16	38	11	1.6 (0.8)	1.8 (1.4)
*Partial drop-outs*^*a*^	204	**24**	**40**	**43**	**25**	**36**	**14**	**2.5 (1.1)**	**1.4 (1.3)**
**Drop-outs, all**	**476**	**26**	**44**	**46**	**25**	**39**	**16**	**2.5 (1.1)**	**1.5 (1.3)**
*Non-responders*	297	23	46	44	24	40	15	2.5 (1.1)	1.5 (1.3)
*Retired*	59	39	37	44	34	36	24	2.4 (1.1)	1.4 (1.1)
*Off duty/change of work*	78	28	47	52	20	39	16	2.5 (1.0)	1.5 (1.4)
*Parental leave*	31	23	45	48	19	32	10	2.4 (1.1)	1.4 (1.3)
*Other reason*^*b*^	11	60	27	46	50	40	27	2.8 (1.1)	1.4 (1.2)

^a^ Partial drop-outs among the occupational categories: Anaesthetic nurses, *N* = 37; Surgical nurses, *N* = 44; Assistant nurses, *N* = 42; Sonographers, *N* = 37; Teachers, *N* = 44

^b^ Excluded since they did not fulfill the inclusion criteria at follow-up (*n* = 8) or all outcome measures missing (*n* = 3)

The questionnaires were administered at baseline (November 2008–October 2012) and at follow-up (November 2011–March 2015), with a mean follow-up period of 28 months (range 20–40 months). The questionnaires at both baseline and at follow-up were sent out to subsets that altered between the various employee categories; i.e. we started with a surgery department, thereafter a school, thereafter some sonography departments, and then another surgery department, and so on. The mean lengths of the follow-up periods were 27 months (range 20–40) for all the nurses, 29 months (20–38) for sonographers and 29 months (range 20–36) for teachers.

### Work tasks

The anaesthetic nurses prepared the patients for surgery, anaesthetized the patient by intubation and checked instruments to ensure that the patient’s general status was maintained during surgery. The surgical nurses were responsible for sterility in the operating theatre and performed, for example, sterile washing of the patient. During surgery, the surgical nurses stood beside the surgeon and assisted with instruments, for example, by holding a surgical retractor to hold the incision open. Assistant nurses assisted other personnel and prepared materials and patients for surgery. For example, they opened a variety of packages containing different materials, moved trolley carts with X-ray equipment, and adjusted the operating lights. All the nurses were involved in turning, lifting and transferring the patient from the gurney to the operating table. The sonographers performed ultrasound examinations of the heart, the blood vessels, or other organs. The sonographers sat or stood at the side of the patient, held a transducer in one hand, operated a keyboard with the other hand and simultaneously observed the image on a screen. After the examination, the sonographers analysed the images at the computer. The teachers taught theoretical subjects to children aged 10–15 years (years 4–9 in the Swedish school system). Additional details concerning the work tasks and the physical workload of the occupational categories studied are given in our previous study [13].

### Questionnaire

The questionnaire included questions about the physical workload, psychosocial working conditions, personal and lifestyle factors, and musculoskeletal pain. Further, the participants were asked about any changes in employment or work tasks during the follow-up period, and whether these changes were associated with musculoskeletal pain. Physical workload: The mechanical exposure index (MEI [29]) comprised 11 items covering work postures and movements, while the physical exposure index (PHYI [29]) included seven items concerned with physical activity and lifting. The questions were answered on a three-point scale in both cases: 1,” little/not at all”; 2,“somewhat”; or 3,“a great deal”. The data were analysed following the classification of Balogh et al. [29]: i.e., the sum of the points was calculated for each scale (MEI = 11–33; PHYI = 7–21), for each individual. The level of mechanical exposure was then divided into four categories: no exposure (11–12 points), low exposure (13–15 points), medium exposure (16–19 points) and high exposure (20–33 points). Physical exposure was similarly divided into no exposure (7–8 points), low exposure (9–10 points), medium exposure (11–13 points) and high exposure (14–21 points). Sensory demands, e.g. eyesight, attention, control of body movements and precision, were measured using a five-item subscale from the Copenhagen Psychosocial Questionnaire [30]. The questions were answered on a five-point scale with the options 1: “to a very large extent”; 2: “to a large extent”; 3: “to some extent”; 4: “to a small extent” and 5: “to a very small extent”, or 1: “always”; 2: “often”; 3: “sometimes”; 4: “seldom” or 5: “never/almost never”. The mean value of the dimension was calculated for each individual.

A study-specific sum score of ergonomic factors, including MEI, PHYI and sensory demands, was then calculated. For MEI and PHYI, each participant was assigned one to four points, corresponding to the categories of mechanical and physical exposure described above (from no exposure to high exposure [29]). For sensory demands, where no predefined cut-offs were available, the study population was divided into quartiles, and the participants in each quartile were assigned one to four points, from the lowest quartile to the highest. In total, the sum of points ranged from 3 to 12. The number of categories was then reduced into the sum score of ergonomic factors (3–6 points = 1; 7–8 points = 2; 9–10 points = 3; and 11–12 points = 4). The separate results for the dimensions MEI, PHYI and sensory demands are given in Additional Table 1 and Additional Table 2.

Satisfaction with conditions during computer work was assessed using the study-specific question: “Are you satisfied with the computer workstation arrangements?”, with the options 1: very satisfied (can work comfortably) or rather satisfied, 2: neither satisfied nor dissatisfied, 3: rather dissatisfied or very dissatisfied (uncomfortable/strenuous work).

Psychosocial working conditions: The psychosocial exposure, in terms of job demands, job control and job support from co-workers, was measured with a Swedish version of the Job Content Questionnaire (JCQ) [31, 32]. Job demands were categorized in terms of nine items, e.g. working pace, hard work, excessive demands, time pressure, conflicting demands and stressful work. Job control included nine items of decision latitude (e.g. influence at work, freedom to decide how work should be done), and skill discretion (e.g. development opportunities, skill and creativity). In the dimension job support, all four items concerning support from co-workers were used. The responses to each item were given on a four-point scale, indicating the level of agreement with various statements about conditions at work. The mean value in each dimension was calculated for each individual. Higher numbers indicate higher demands, better control, and better support. A subset of the Copenhagen Psychosocial Questionnaire [30] was used to measure emotional demands (three items concerning e.g. emotionally difficult situations and emotional effects of work), demands on hiding emotions (two items), and leadership (eight items concerning planning of work, conflict solving, communication and concern for staff). All questions were answered on a five-point scale with the options 1: “to a very large extent”; 2: “to a large extent”; 3: “to some extent”; 4: “to a small extent” and 5: “to a very small extent”, or 1: “always”; 2: “often”; 3: “sometimes”; 4: “seldom” or 5: “never/almost never”. The mean value was calculated for each dimension for each individual.

A study-specific sum score was calculated for the psychosocial factors, based on the six dimensions: job demands, job control, job support from colleagues, emotional demands, demands of hiding emotions and leadership. For each of the dimensions job demands, emotional demands and demands of hiding emotions, the individuals in the upper quartile of the study population were assigned one point, and the remaining participants zero points. For the dimensions job control, job support and leadership, the individuals in the lowest quartile of the population were assigned one point and the remaining participants zero points. A sum score of 0–6 possible points was calculated for each individual. Due to few individuals with five or six points these two groups were combined, resulting in a possible sum score of psychosocial factors of 0–5. The separate results for the six dimensions included in the sum score are given in additional Table 1 and Additional Table 2.

The occupational category for each individual was included in the analysis, in order to explain any predictive factors for pain, that were not covered by the questionnaire comprising questions about ergonomic, psychosocial and personal factors.

Personal and lifestyle factors: The participants were asked to give their age, seniority, height and weight. Their body mass index (BMI; kg/m^2^) was then calculated. They were also asked about personal relaxation [33]: “How much of your leisure time (except weekends/holidays) do you usually spend for your own relaxation (without special requirements and obligations)?” (1: ≥3 h/day; 2: 1–2 h/day; and 3: < 1 h/day), domestic work [33]: “How many hours a week do you spend working in the home that is not paid work, e.g. shopping, cooking, taking care of finances, washing, cleaning, caring for children, maintaining a car, house and garden?” (1: < 10 h/week; 2: 11–20 h/week; 3: > 21 h/week), physical exercise [33]: “Do you spend your leisure time exercising in any way? Exercise includes sports, fitness training, gymnastics, dancing, walking, cycling, etc., for at least 30 minutes per occasion” (1: twice a week or more; 2: once a week; 3: occasionally or never); and smoking habits (0: never smoked; 1: ex-smoker of at least 6 months’ standing; 2: smoker, but not daily; 3: daily smoker). In the analysis, the smoking categories 0–2 were merged into one category of “not daily smokers”.

Musculoskeletal pain [13]: The participants were asked about subjective musculoskeletal complaints at nine anatomical sites: the neck, shoulders, elbows, hands, upper back, lower back, hips, knees and feet, during the preceding 12 months, according to the Standardized Nordic Questionnaire for the Analysis of musculoskeletal Symptoms [34]. For the shoulders, elbows, hands, hips, knees and feet, pain in one or both sides of the body was regarded as one pain site. In addition, information was collected for each anatomical site on the frequency of complaints during the past year using a 5-point scale (never, seldom, sometimes, often, or very often [35]), as well as the intensity of complaints on an eleven-point scale, from 0 (none at all) to 10 (very, very severe [36]). The individual was considered to have considerable musculoskeletal pain (subsequently referred to simply as “pain”) if reporting complaints at least “seldom” with an intensity of at least 7 (very severe), or “sometimes” with an intensity of at least 3 (moderate), or “often” or “very often” with an intensity of at least 2 (slight/mild) [13]. The condition was defined separately for each anatomical site.

The number of anatomical sites with pain was calculated for each individual (0–9). For assessment of multisite pain, we followed the suggested classification by Pereira de Fernandes and Burdorf [26]: The participants were divided into five categories: 0, no pain; 1, one pain site; 2, two pain sites; 3, three pain sites and 4, ≥ four pain sites. Furthermore, five of the anatomical regions were selected for analysis of specific pain sites: the neck, shoulders, hands, lower back and feet.

### Statistical analyses

All statistical analyses were performed with IBM SPSS software, version 24 (IBM Corp.). *P*-values ≤0.05 (two-tailed) were considered statistically significant. Differences between the prevalence of pain at baseline and at follow-up were evaluated with the McNemar test. Prevalence ratios (PRs) and 95% confidence intervals (CIs) for pain in the neck, shoulders, hands, lower back and feet at follow-up were first estimated in single-exposure Poisson regression models, with unit length of follow-up, for all variables collected at baseline (in total eleven factors including pain at the specific anatomical site, sum scores of ergonomic and psychosocial factors, computer work, age, BMI, personal relaxation, domestic work, physical exercise, smoking and occupational category). In the next step, PRs for pain at follow-up were estimated using Poisson regression, with unit length of follow-up, with multiple exposures (multi-exposure model), without pain at baseline. In the third step, by adjusting the multi-exposure model for pain at baseline we tried to quantify how much of the explanation from different factors concerning pain at follow-up that was not due to associations with pain that were present already at baseline. Due to the high collinearity (strong correlation) between seniority and age, seniority was omitted from the multi-exposure statistical analysis.

For multisite pain at follow-up (i.e. number of pain sites stratified into five categories: 0, 1, 2, 3 and ≥ 4 sites), associations with the eleven occupational and personal factors collected at baseline were first investigated using single-exposure ordinal regression models under the cumulative odds model with location parameters only [37, 38]. This model estimates the average odds ratios (ORs) and 95% CIs of all possible dichotomisations of the ordinal response variable. The importance of the number of pain sites pain at baseline, for the number of pain sites at follow-up, was also estimated separately. Next, ORs for the number of pain sites at follow-up were estimated using multi-exposure ordinal regression, without the number of pain sites at baseline. Finally, the multi-exposure models were adjusted for the number of pain sites at baseline. In all regression analyses, the overall *p*-value reflects the strength of the empirical evidence that the pain prevalence is not constant across the contrasted groups.

As supplementary analyses we also fitted single- and multi-exposure models with the separate dimensions included in the sum-scores of ergonomic and psychosocial factors, for all pain outcomes.

## Results

The distributions of musculoskeletal pain and occupational factors at baseline were generally similar among participants and drop-outs (*n* = 476) at follow-up, with two exceptions: Compared to the participants, the drop-outs had a higher frequency of neck pain (44% vs. 39%), and a higher sum score of psychosocial factors [mean 1.5 (SD 1.3) vs. mean 1.3 (SD 1.2)]. The results are given in Table 1.

The effective number of participants varied between 911 (in the multi-exposure model of multisite pain, adjusted for the number of pain sites at baseline) and 1115 (for age groups and occupational groups), due to missing data for some variables, referred to as “partial drop-outs”. The major variables for partial drop-outs were the sum score of ergonomic factors (*n* = 69), psychosocial factors (*n* = 45), number of pain sites at follow-up (*n* = 56) and satisfaction with the computer workstation arrangement (*n* = 50). The number of partial drop-outs for the number of pain sites at baseline was 21.

At follow-up, the distribution of the number of pain sites in the total population was as follows: Zero pain sites, 21,2% (*N* = 224); one pain site, 17,6% (*N* = 186); two pain sites, 17,1% (*N* = 181); three pain sites, 16,4% (*N* = 174); four pain sites, 11,2% (*N* = 119); five pain sites, 7,5% (*N* = 79); six pain sites, 5,4% (*N* = 57); seven pain sites, 2,3% (*N* = 24); eight pain sites, 0,9% (*N* = 10); and nine pain sites 0,5% (*N* = 5).

### Changes in the presence of pain between baseline and follow-up

**Fig. 1:**
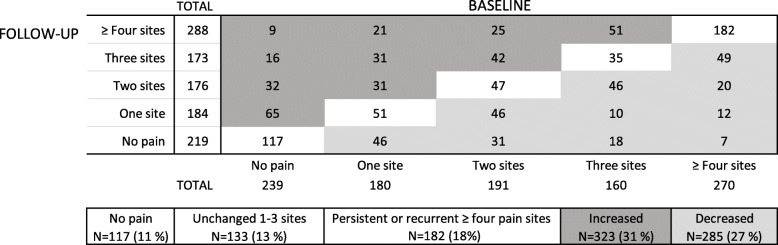
Cross-tabulation of the number of anatomical sites at which pain was reported by the participants at baseline and at follow-up (*n* = 1040)

Pain at four or more anatomical sites, was reported by 26% of the participants at baseline vs. 28% at follow-up (*p* = 0.22). Among these, 182 participants (18% of the study population) reported pain at four or more anatomical sites on both occasions (Fig. 1). Eleven percent (117 individuals) did not report pain at any site, either at baseline or at follow-up. The number of pain sites differed between baseline and follow-up in the majority of the participants, in both directions: 31% reported pain at more sites at follow-up, while 27% reported pain at fewer sites.

The prevalence of neck pain in the total study population increased between baseline and follow-up (39% vs. 42%; *p* = 0.05; data not given in Table 1), while no statistically significant differences were seen between the two time-points for other anatomical sites. The surgical nurses reported a higher frequency of neck pain at follow-up than at baseline (47% vs. 39%; *p* = 0.03). The prevalence of lower back pain among anaesthetic nurses decreased at follow-up (30% vs. 39%; *p* = 0.03), while it increased among the sonographers (39% vs. 28%; *p* = 0.001). The teachers reported a higher frequency of pain in the feet at follow-up (18% vs. 11% at baseline; *p* = 0.01; data not given).

### Associations between specific ergonomic and psychosocial factors and musculoskeletal pain

Results from single- and multi-exposure models between the separate dimensions included in the sum-scores of ergonomic and psychosocial factors at baseline, and musculoskeletal pain (number of pain sites and the five specific pain outcomes) at follow-up, are reported in Additional Table 1 and Additional Table 2, respectively. In the multi-exposure models (adjusted for all personal- and life-style factors), there were statistically significant associations between high values of mechanical exposure index at baseline and the number of pain sites at follow-up (Additional Table 2). Mechanical exposure index was also associated with pain in the neck, shoulders, and lower back at follow-up.

Among the specific psychosocial dimensions, few consistent associations were observed across the various outcomes. High job demands at baseline was associated with pain in the lower back at follow-up, and low job control was associated with shoulder pain (Additional Table 2). High emotional demands and high demands of hiding emotions at baseline were associated with the number of pain sites at follow-up. High demands of hiding emotions was also associated with hand pain at follow-up.

### Risk factors for several sites of pain

**Fig. 2:**
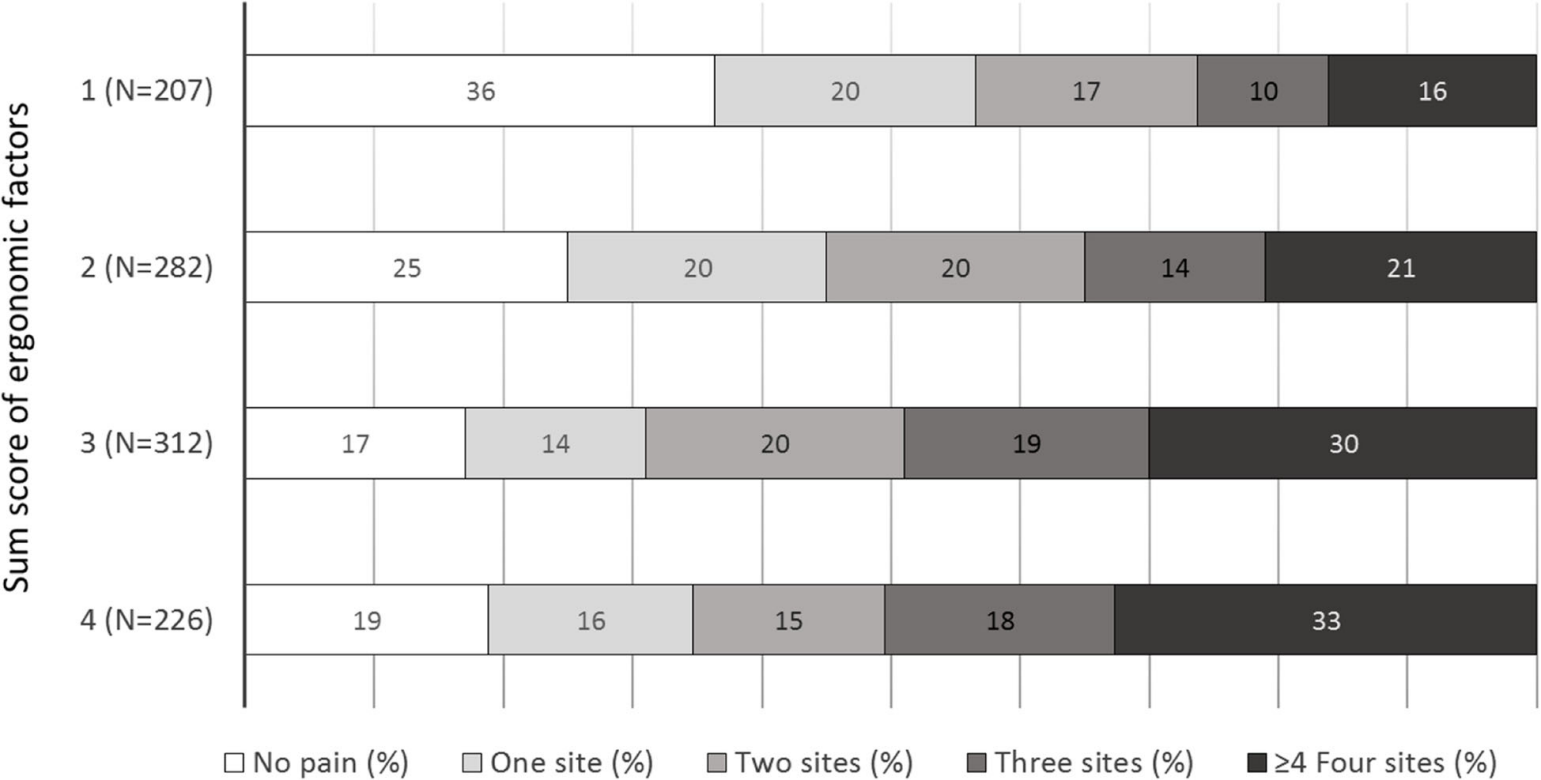
The association between the sum score for ergonomic factors (1–4) at baseline and the number of anatomical sites with pain at follow-up

**Fig. 3:**
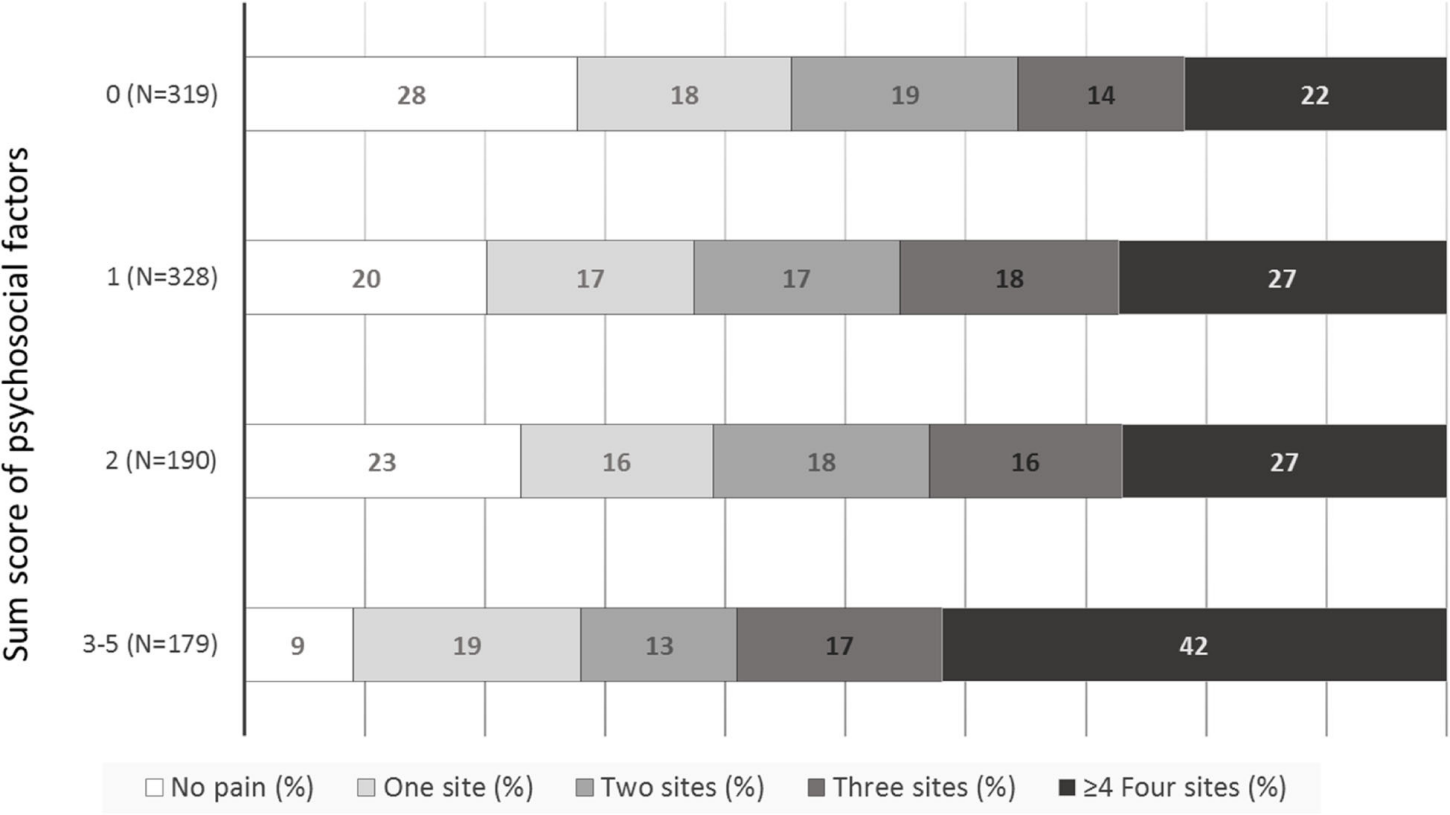
The association between the sum score for psychosocial factors (0, 1, 2 and 3–5) at baseline and the number of anatomical sites with pain at follow-up

Occupational exposures, i.e. the sum scores of ergonomic and psychosocial factors, were risk factors for a high number of pain sites at follow-up, with statistically significant associations in both the single- multi- and adjusted multi-exposure models (Table 2). The associations between the sum score for ergonomic factors (1–4) and the sum score for psychosocial factors (0, 1, 2 and 3–5) at baseline, and the number of anatomical sites with pain at follow-up, are shown in Fig. 2 and Fig. 3, respectively.

**Table 2:** Single- and multi-exposure ordinal regression models in the total study population, of associations between self-reported ergonomic, psychosocial and personal factors at baseline and musculoskeletal pain (number of pain sites in five categories) at follow-up, calculated with ordinal regression with overall *p*-values, odds ratios (ORs)^a^ and 95% confidence intervals (CIs). In the last step, in a complete case analysis (*N* = 911), the multi-exposure model was adjusted for the number of pain sites at baseline. Results in bold face are statistically significant

	Single exposure			Multi-exposure (*N* = 925)		Multi-exposure, adjusted (*N* = 911)	
	N	*p*	OR (CI)	*p*	OR (CI)	*p*	OR (CI)
Pain at baseline^b^	1040	**< 0.001**			–	**< 0.001**	
*(Number of pain sites)*							
*0*	239		1		–		1
*1*	180		**2.89 (2.02–4.13)**		–		**2.53 (1.70–3.74)**
*2*	191		**4.44 (3.13–6.32)**				**3.99 (2.72–5.86)**
*3*	160		**10.8 (7.32–15.9)**		–		**10.6 (6.95–16.2)**
≥ 4 pain sites	270		**49.1 (33.3–72.6)**		–		**42.9 (27.8–66.4)**
Sum score of ergonomic factors (*scale*) ^b^	991	**< 0.001**		**< 0.001**		**0.01**	
*1*	198		1		1		1
*2*	264		**1.48 (1.07–2.07)**		1.36 (0.92–2.00)		1.17 (0.79–1.74)
*3*	304		**2.43 (1.75–3.36)**		**2.09 (1.40–3.13)**		**1.55 (1.02–2.35)**
*4*	225		**2.64 (1.86–3.73)**		**2.20 (1.39–3.46)**		**1.67 (1.04–2.70)**
Complaints about computer workstation arrangements^b^	1011	0.14		0.23		0.93	
*Satisfied*	436		1		1		1
*Neutral*	329		1.22 (0.95–1.57)		1.36 (1.03–1.81)		1.17 (0.87–1.57)
*Dissatisfied*	246		1.20 (0.91–1.59)		1.19 (0.87–1.64)		1.02 (0.73–1.43)
Sum score of psychosocial factors (*scale*) ^b^	1016	**< 0.001**		**< 0.001**		**0.02**	
*0*	319		1		1		1
*1*	328		**1.46 (1.11–1.92)**		**1.40 (1.04–1.88)**		1.27 (0.94–1.73)
*2*	190		1.36 (0.99–1.87)		1.37 (0.97–1.96)		1.00 (0.69–1.45)
*3*	120		**2.81 (1.91–4.14)**		**2.86 (1.88–4.35)**		**2.02 (1.29–3.14)**
*4*	44		**2.51 (1.44–4.35)**		**3.31 (1.73–6.33)**		1.85 (0.91–3.77)
*5*	15		2.09 (0.84–5.17)		**2.84 (1.00–8.01)**		1.37 (0.42–4.48)
Age group (*years*)^c^	1059	0.44		0.62		0.90	
*< 40*	262		1		1		**1**
*40–55*	515		1.18 (0.91–1.54)		1.12 (0.83–1.50)		1.02 (0.75–1.40)
*> 55*	282		1.15 (0.85–1.55)		1.19 (0.84–1.68)		1.09 (0.75–1.58)
Body Mass Index (*points*) ^c^	1038	**0.003**		**0.03**		0.41	
*< 18.5*	11		1.13 (0.45–2.88)		1.29 (0.46–3.64)		1.18 (0.38–3.61)
*18.5–24.9*	673		1		1		1
*25.0–29.9*	271		1.28 (1.00–1.64)		1.29 (0.98–1.70)		0.95 (0.71–1.27)
*> 30*	83		**2.06 (1.37–3.12)**		**1.84 (1.18–2.89)**		1.44 (0.91–2.29)
Time for personal relaxation^b^	1039	0.14		0.55		0.91	
*≥ 3 h /day*	265		1		1		1
*1–2 h/day*	547		1.16 (0.90–1.51)		1.33 (0.99–1.79)		1.16 (0.85–1.58)
*< 1 h/day*	227		1.26 (0.93–1.72)		1.10 (0.76–1.61)		1.02 (0.68–1.52)
Domestic work^b^	1046	0.68		0.63		0.17	
*0–10 h/week*	360		1		1		1
*11–20 h/week*	435		0.84 (0.65–1.07)		**0.74 (0.56–0.97)**		**0.72 (0.54–0.96)**
*≥ 21 h/week*	251		1.10 (0.83–1.46)		0.96 (0.69–1.33)		0.82 (0.58–1.15)
Physical exercise^b^	1051	**0.02**		0.63		0.15	
*Twice a week or more*	*764*		1		1		1
*Once a week*	*146*		1.30 (0.95–1.78)		1.12 (0.79–1.58)		1.33 (0.92–1.92)
*Occasionally or never*	*141*		**1.39 (1.01–1.91)**		1.09 (0.74–1.60)		1.24 (0.83–1.87)
Daily smokers	1055	0.44		0.20		0.27	
*no*	1011		1		1		1
*yes*	44		1.24 (0.73–2.10)		0.67 (0.37–1.23)		0.70 (0.37–1.32)
Occupational category ^c^	1059	**< 0.001**		**0.001**		**0.02**	
*Teacher*	229		1		1		1
*Anaesthetic nurse*	206		0.92 (0.66–1.29)		0.77 (0.50–1.18)		0.73 (0.47–1.14)
*Surgical nurse*	197		**1.83 (1.30–2.57)**		1.31 (0.82–2.09)		1.31 (0.80–2.14)
*Assistant nurse*	213		**1.82 (1.30–2.55)**		1.27 (0.81–1.97)		1.06 (0.67–1.68)
*Sonographer*	214		**1.71 (1.22–2.39)**		**1.72 (1.14–2.61)**		1.40 (0.90–2.16)

^a^ The average ORs and 95% CIs of all possible dichotomisations of the ordinal response variable

^b^ Trend *p*-value obtained by using the categorical exposure variable as continuous in the analysis

^c^ Overall *p*-value for the categorical exposure variable

Among the personal factors, a high BMI and a low frequency of physical exercise were of importance in the single-exposure models (Table 2). A high BMI was also a risk factor in the multi-exposure model, but when the model was adjusted for the number of pain sites at baseline, the importance of a high BMI disappeared. A high number of pain sites at baseline was the strongest risk factor for a high number of pain sites at follow-up. Furthermore, a residual of unknown factors associated with the occupational category remained, which were of importance for a high number of pain sites, in all three statistical models.

### Single-exposure models of risk factors for pain in specific sites

Results from the single-exposure models, for the five specific anatomical sites (the neck, shoulders, hands, lower back and feet), are given in Additional Table 3. The main results obtained from these models were that high sum scores of both ergonomic and psychosocial factors at baseline were statistically significant risk factors for pain in the neck, shoulders, hands and the lower back at follow-up. A high sum score for ergonomic factors was also a risk factor for pain in the feet. Additional factors that were of importance for specific pain sites at follow-up were complaints associated with the arrangement of the computer workstation (pain in the neck), increased age (pain in the hands and feet), a high BMI (pain in the feet), lack of time for personal relaxation (pain in the neck and shoulders), a low frequency of physical exercise (pain in the neck and in the lower back) and occupational category (all five anatomical sites). However, having pain at a specific anatomical site at baseline was the strongest predictor of pain at the same site at follow-up, with PRs ranging from 2.7 to 4.7 between the sites. Much domestic work was a protective factor for hand pain and daily smoking was a protective factor for neck pain.

### Multi-exposure models of risk factors for pain in specific sites

All results in this section are given in Table 3 (unadjusted multi-exposure model) and in Table 4 (multi-exposure model adjusted for pain in the same anatomical site at baseline).

**Table 3:** Multi-exposure models in the total study population (*n* = 1115) of associations between self-reported ergonomic, psychosocial and personal factors at baseline, and musculoskeletal pain in the neck, shoulders, hands, lower back and feet at follow-up, calculated with Poisson regression, with overall *p*-values, prevalence ratios (PRs) and 95% confidence intervals (CIs). Results in bold face are statistically significant

		Neck (*N* = 959)		Shoulders (*N* = 950)		Hands (*N* = 966)		Lower back (*N* = 959)		Feet (*N* = 967)	
	N	*p*	PR (CI 95%)	*p*	PR (CI 95%)	*p*	PR (CI 95%)	*p*	PR (CI 95%)	*p*	PR (CI 95%)
Sum score of ergonomic factors (*scale*)^a^	1046	**< 0.01**		**0.02**		**0.001**		0.25		**0.02**	
*1*	211		1		1		1		1		1
*2*	283		1.13 (0.88–1.47)		0.97 (0.76–1.23)		1.04 (0.70–1.56)		1.28 (0.97–1.69)		1.14 (0.70–1.84)
*3*	321		**1.30 (1.01–1.69)**		1.24 (0.99–1.57**)**		**1.69 (1.16–2.46)**		**1.45 (1.09–1.91)**		1.32 (0.80–2.17)
*4*	231		**1.55 (1.16–2.06)**		1.24 (0.95–1.61)		**1.65 (1.09–2.51)**		1.23 (0.89–1.69)		**1.75 (1.03–2.98)**
Complaints about computer workstation arrangements^a^	1065	**0.02**		0.05		0.44		0.31		0.38	
*Satisfied*	463		1		1		1		1		1
*Neutral*	341		**1.27 (1.06–1.52**)		1.12 (0.94–1.32)		1.03 (0.81–1.32)		1.25 (1.04–1.49)		1.20 (0.88–1.63)
*Dissatisfied*	261		**1.25 (1.03–1.52)**		**1.19 (1.00–1.42)**		0.92 (0.69–1.23)		1.06 (0.86–1.32)		0.85 (0.58–1.24)
Sum score of psychosocial factors (*scale*) ^a^	1070	**< 0.001**		**< 0.001**		**0.03**		**0.003**		0.66	
*0*	337		1				1		1		1
*1*	339		**1.23 (1.00–1.50)**		1.02 (0.85–1.23)		**1.43 (1.08–1.88)**		1.13 (0.92–1.39)		1.30 (0.94–1.80)
*2*	203		**1.28 (1.02–1.61)**		1.17 (0.96–1.43)		1.01 (0.71–1.45)		1.23 (0.98–1.55)		0.84 (0.54–1.31)
*3*	130		**1.41 (1.11–1.78)**		**1.36 (1.10–1.69)**		**1.65 (1.19–2.29)**		**1.38 (1.08–1.77)**		1.16 (0.75–1.79)
*4*	*45*		**1.67 (1.20–2.32)**		**1.64 (1.24–2.16)**		1.37 (0.81–2.31)		**1.57 (1.11–2.23)**		0.59 (0.21–1.65)
*5*	*16*		**1.93 (1.26–2.95)**		**1.78 (1.19–2.67)**		1.79 (0.91–3.50)		1.42 (0.84–2.39)		1.49 (0.70–3.18)
Age group (*years*)^b^	1115	0.85		0.50		**0.01**		0.75		**< 0.01**	
*< 40*	267		1		1		1		1		1
*40–55*	544		0.95 (0.80–1.14)		0.93 (0.79–1.10)		1.14 (0.86–1.53)		1.08 (0.88–1.32)		**2.34 (1.45–3.76)**
*> 55*	304		0.99 (0.80–1.22)		0.89 (0.73–1.09)		**1.54 (1.12–2.10)**		1.03 (0.82–1.31)		**2.20 (1.31–3.71)**
Body Mass Index (*points*)^b^	1092	0.92		0.12		0.20		0.37		**< 0.01**	
*< 18.5*	11		1.05 (0.48–2.32)		0.89 (0.43–1.81)		1.55 (0.60–4.01)		0.70 (0.28–1.73)		1.20 (0.18–8.00)
*18.5–24.9*	707		1		1		1		1		1
*25.0–29.9*	287		1.06 (0.90–1.25)		**1.18 (1.01–1.36)**		0.96 (0.75–1.23)		1.03 (0.86–1.23)		1.17 (0.86–1.60)
*> 30*	87		1.02 (0.78–1.33)		1.20 (0.94–1.53)		1.36 (0.98–1.88)		1.21 (0.95–1.55)		**1.90 (1.32–2.75)**
Time for personal relaxation^a^	1094	**< 0.01**		**0.01**		0.34		0.97		0.81	
*≥ 3 h /day*	281		1		1		1		1		1
*1–2 h/day*	577		**1.27 (1.04–1.55)**		**1.27 (1.05–1.53)**		1.12 (0.87–1.44)		1.06 (0.875–1.28)		1.29 (0.93–1.79)
*< 1 h/day*	236		**1.36 (1.07–1.74)**		**1.35 (1.08–1.70)**		1.18 (0.83–1.67)		1.0 (0.77–1.285)		0.98 (0.62–1.57)
Domestic work^a^	1102	0.96		0.96		0.07		0.85		0.07	
*0–10 h/week*	382		1		1		1		1		1
*11–20 h/week*	459		0.87 (0.73–1.04)		0.89 (0.76–1.05)		0.77 (0.61–0.97)		0.96 (0.80–1.15)		0.78 (0.58–1.06)
*≥ 21 h/week*	261		1.02 (0.84–1.24)		1.00 (0.83–1.20)		0.78 (0.58–1.04)		0.99 (0.80–1.22)		0.71 (0.49–1.03)
Physical exercise^a^	1107	0.83		0.30		0.48		0.56		0.29	
*Twice a week or more*	807		1		1		1		1		1
*Once a week*	153		0.98 (0.80–1.20)		0.95 (0.79–1.15)		0.88 (0.65–1.20)		1.05 (0.85–1.29)		0.79 (0.52–1.18)
*Occasionally or never*	147		0.99 (0.79–1. 23)		0.90 (0.71–1.13)		0.92 (0.64–1.30)		1.05 (0.84–1.31)		0.87 (0.56–1.33)
Daily smokers	1111	**0.02**		0.26		0.61		0.80		0.18	
*no*	1063		1		1		1		1		1
*yes*	48		**0.57 (0.35–0.93)**		0.80 (0.54–1.18)		0.87 (0.51–1.49)		1.05 (0.72–1.53)		0.64 (0.33–1.23)
Occupational category ^b^	1115	**< 0.001**		**< 0.001**		**0.03**		**0.05**		**0.05**	
*Teacher*	246		**1**		**1**		1		1		1
*Anaesthetic nurse*	214		0.74 (0.56–0.98)		1.05 (0.81–1.37)		0.92 (0.59–1.41)		0.78 (0.57–1.05)		0.75 (0.46–1.24)
*Surgical nurse*	209		0.99 (0.75–1.31)		1.13 (0.86–1.49)		1.16 (0.76–1.77)		1.19 (0.89–1.58)		0.77 (0.47–1.25)
*Assistant nurse*	224		0.93 (0.70–1.22)		1.12 (0.86–1.45)		**1.50 (1.03–2.19)**		1.10 (0.84–1.44)		0.86 (0.55–1.36)
*Sonographer*	222		**1.29 (1.02–1.63)**		**1.69 (1.35–2.11)**		1.38 (0.94–2.02)		1.07 (0.82–1.39)		**0.43 (0.24–0.76)**

^a^ Trend *p*-value obtained by using the categorical exposure variable as continuous in the analysis

^b^ Overall *p*-value for the categorical exposure variable

**Table 4:** Multi-exposure models adjusted for pain at baseline in the total study population (*n* = 1115) of associations between self-reported ergonomic, psychosocial and personal factors at baseline, and musculoskeletal pain in the neck, shoulders, hands, lower back and feet at follow-up, calculated with Poisson regression, with overall *p*-values, prevalence ratios (PRs) and 95% confidence intervals (CIs). Results in bold face are statistically significant

		Neck (*N* = 956)		Shoulders (*N* = 945)		Hands (*N* = 963)		Lower back (*N* = 956)		Feet (*N* = 966)	
	N	*p*	PR (CI 95%)	*p*	PR (CI 95%)	*p*	PR (CI 95%)	*p*	PR (CI 95%)	*p*	PR (CI 95%)
Pain at baseline^a^		**< 0.001**		**< 0.001**		**< 0.001**		**< 0.001**		**< 0.001**	
*no*			1		1		**1**		**1**		**1**
*yes*			**3.01 (2.55–3.56)**		**2.42 (2.07–2.84)**		**3.49 (2.83–4.32)**		**2.75 (2.34–3.24)**		**4.22 (3.25–5.47)**
Sum score of ergonomic factors (*scale*)^b^	1046	**0.04**		0.11		**< 0.01**		0.46		**0.04**	
*1*	211		1		1		1		1		1
*2*	283		1.10 (0.87–1.39)		0.94 (0.75–1.18)		1.01–0.69-1.47)		1.20 (0.94–1.53)		1.04 (0.66–1.64)
*3*	321		1.15 (0.91–1.46)		1.09 (0.88–1.35)		**1.54 (1.08–2.20)**		**1.29 (1.01–1.64)**		1.18 (0.75–1.86)
*4*	231		**1.33 (1.01–1.75)**		1.14 (0.88–1.46)		1.44 (0.96–2.16)		1.13 (0.85–1.51)		1.64 (0.96–2.79)
Complaints about computer workstation arrangements^b^	1065	0.19		0.39		0.21		0.30		0.52	
*Satisfied*	463		1		1		1		1		1
*Neutral*	341		1.16 (0.98–1.36)		1.03 (0.88–1.2)		1.05 (0.83–1.32)		1.20 (1.02–1.42)		1.16 (0.88–1.53)
*Dissatisfied*	261		1.13 (0.95–1.35)		1.07 (0.92–1.25)		0.86 (0.66–1.13)		1.08 (0.88–1.31)		0.88 (0.61–1.27)
Sum score of psychosocial factors (*scale*) ^b^	1070	**0.01**		**0.001**		0.48		0.13		0.10	
*0*	337		1		1		1		1		1
*1*	339		1.16 (0.97–1.38)		1.02 (0.87–1.21)		**1.35 (1.05–1.73)**		1.03 (0.85–1.24)		1.09 (0.80–1.48)
*2*	203		1.13 (0.92–1.39)		1.12 (0.93–1.35)		0.92 (0.67–1.27)		1.06 (0.85–1.31)		0.69 (0.46–1.03)
*3*	130		1.16 (0.94–1.44)		**1.25 (1.02–1.52)**		1.32 (0.96–1.81)		1.23 (0.98–1.55)		0.89 (0.58–1.36)
*4*	*45*		1.35 (0.99–1.82)		**1.50 (1.13–2.0)**		1.10 (0.67–1.8)		1.27 (0.93–1.73)		0.53 (0.20–1.36)
*5*	*16*		**2.29 (1.43–3.66**)		**1.56 (1.08–2.24)**		1.34 (0.71–2.54)		0.97 (0.58–1.64)		0.94 (0.46–1.91)
Age group (*years*)^c^	1115	0.69		0.59		0.42		0.54		**0.02**	
*< 40*	267		**1**		**1**		**1**		**1**		**1**
*40–55*	544		0.96 (0.82–1.13)		0.92 (0.79–1.08)		1.03 (0.78–1.37)		1.09 (0.90–1.32)		**1.94 (1.21–3.12)**
*> 55*	304		1.03 (0.85–1.25)		0.95 (0.79–1.14)		1.19 (0.87–1.63)		1.01 (0.82–1.25)		**1.79 (1.08–2.96)**
Body Mass Index (*points*)^c^	1092	0.96		0.55		0.26		0.58		0.35	
*< 18.5*	11		0.83 (0.39–1.78)		0.97 (0.41–2.32)		1.77 (0.70–4.47)		0.76 (0.34–1.68)		1.36 (0.29–6.46)
*18.5–24.9*	707		**1**		**1**		**1**		**1**		1
*25.0–29.9*	287		0.99 (0.86–1.15)		1.08 (0.95–1.24)		0.95 (0.75–1.2)		1.03 (0.87–1.22)		0.95 (0.70–1.29)
*> 30*	87		1.03 (0.81–1.31)		1.14 (0.91–1.42)		1.26 (0.94–1.71)		1.14 (0.92–1.43)		1.31 (0.92–1.86)
Time for personal relaxation^b^	1094	**0.02**		0.15		0.31		0.62		0.78	
*≥ 3 h /day*	281		1		1		1		1		1
*1–2 h/day*	577		1.19 (0.99–1.43)		1.12 (0.94–1.33)		1.06 (0.84–1.35)		1.0 (0.84–1.18)		1.24 (0.91–1.70)
*< 1 h/day*	236		**1.29 (1.03–1.61)**		1.18 (0.95–1.46)		1.16 (0.84–1.59)		0.95 (0.76–1.19)		1.02 (0.65–1.60)
Domestic work^b^	1102	0.44		0.82		**0.03**		0.44		**0.03**	
*0–10 h/week*	382		1		1		1		1		1
*11–20 h/week*	459		0.87 (0.75–1.02)		0.92 (0.79–1.07)		**0.78 (0.63–0.96)**		0.94 (0.81–1.11)		0.79 (0.59–1.04)
*≥ 21 h/week*	261		0.95 (0.80–1.14)		0.98 (0.83–1.17)		**0.74 (0.55–0.99)**		0.94 (0.77–1.14)		**0.66 (0.46–0.93)**
Physical exercise^b^	1107	0.94		0.57		0.50		0.55		0.17	
*Twice a week or more*	807		1		1		1		1		1
*Once a week*	153		1.01 (0.85–1.21)		0.98 (0.83–1.17)		0.96 (0.72–1.26)		1.04 (0.85–1.26)		0.81 (0.56–1.18)
*Occasionally or never*	147		1.01 (0.82–1.25)		0.95 (0.77–1.16)		0.88 (0.63–1.22)		1.03 (0.84–1.27)		0.79 (0.52–1.21)
Daily smokers	1111	**0.02**		0.17		0.43		0.34		0.12	
*no*	1063		1		1		1		1		1
*yes*	48		**0.59 (0.37–0.92)**		0.77 (0.53 (1.12)		0.83 (0.53–1.31)		1.15 (0.86–1.53)		0.63 (0.36–1.13)
Occupational category ^c^	1115	**0.02**		**< 0.001**		0.39		**0.01**		**0.02**	
*Teacher*	246		**1**		**1**		1		1		1
*Anaesthetic nurse*	214		0.86 (0.66–1.11)		1.03 (0.81–1.32)		0.89 (0.59–1.35)		0.8 (0.60–1.07)		0.65 (0.40–1.06)
*Surgical nurse*	209		1.08 (0.83–1.41**)**		1.08 (0.84–1.39)		1.03 (0.7–1.53)		1.20 (0.93–1.56)		0.68 (0.42–1.09)
*Assistant nurse*	224		0.99 (0.76–1.29)		1.07 (0.84–1.36)		1.19 (0.83–1.70)		1.08 (0.85–1.38)		0.72 (0.48–1.08)
*Sonographer*	222		1.21 (0.97–1.5)		**1.44 (1.18–1.78)**		1.18 (0.84–1.67)		1.22 (0.95–1.57)		**0.40 (0.23–0.69)**

^a^ The frequency of musculoskeletal pain in the different body regions is given in Table 1

^b^ Trend *p*-value obtained by using the categorical exposure variable as continuous in the analysis

^c^ Overall *p*-value for the categorical exposure variable

The sum score for ergonomic factors at baseline was associated with pain in the neck, shoulders, hands and feet at follow-up. When adjusting the multi-exposure models for pain in the specific anatomical site at baseline, adverse ergonomic factors remained a significant risk factor for pain in the neck, hands and feet. Inadequate computer workstation arrangement was a risk factor for pain in the neck and the shoulders, but these associations declined in the adjusted multi-exposure model. The sum score for psychosocial factors at baseline was associated with pain in the neck, shoulders, hands and the lower back. When adjusting for pain at baseline, the sum score of psychosocial factors remained a statistically significant risk factor for pain in the neck and the shoulders.

High age was a risk factor for pain in both the hands and the feet at follow-up. However, when adjusting for pain at baseline, the statistically significant association remained only for feet pain. A high BMI was associated with pain in the feet in the multi-exposure model, but the association was no longer statistically significant after adjustment for foot pain at baseline. Lack of time for personal relaxation was a risk factor for pain in the neck and the shoulders, but in the adjusted multi-exposure model, it remained significant for neck pain only. Among the other life-style factors, neither a low frequency of physical exercise nor much domestic work predicted pain at any anatomical site. On the contrary, much domestic work was a protective factor for pain in the hands and the feet. Furthermore, daily smoking at baseline was a protective factor for neck pain in both multi-exposure models.

For all of the specific anatomical sites, pain at baseline was the strongest risk factor for pain at that anatomical site at follow-up, with PRs ranging from 2.4 to 4.2 between the sites. After adjustment of all the occupational and personal factors studied, and pain at baseline, a residual of unknown factors associated with the occupational category remained, which were of importance for all of the anatomical pain sites at follow-up. Regarding the specific occupational categories*,* it was observed that there was an increased risk of shoulder pain, but a decreased risk of pain in the feet among sonographers, when pain at baseline was taken into account.

## Discussion

### Principal findings

Adverse ergonomic and psychosocial factors at baseline were risk factors for a high number of pain sites at follow-up, with statistically significant associations in the single-, multi- and adjusted multi-exposure models. However, the strongest risk factor for a high number of pain sites at follow-up was a high number of pain sites at baseline. Furthermore, unknown factors associated with occupational category were found to be of importance for a high number of pain sites in all three statistical models.

In the adjusted multi-exposure models, different risk factors were found for subsequent pain in the five anatomical regions. A high sum score of ergonomic factors at baseline was a risk factor for pain in the neck, hands and feet. A high sum score for psychosocial factors was associated with pain in the neck and shoulders at follow-up. Moreover, lack of time for personal relaxation was a risk factor for neck pain, and high age was a risk factor for pain in the feet. Unexpected findings were that much domestic work was a protective factor for hand and foot pain, and that daily smoking was a protective factor for neck pain. For all the specific anatomical sites, the strongest risk factor for pain at follow-up was pain at the same site at baseline. Furthermore, the occupational category was of importance for pain in the neck, shoulders, lower back and feet at follow-up.

Several of the relationships found in the multi-exposure analyses became less strong when the models were adjusted for pain at baseline. For example, while there was an overall pattern of a relationship between a high number of pain sites and the sum score of psychosocial factors, the ORs for some of the categories decreased in the adjusted model. This indicates that an important part of the associations was present already at baseline.

### Strengths and weaknesses of the study

Strengths of this study are the longitudinal study design, and the fact that we used common and tested indicators for physical and psychosocial working conditions. Another advantage is the outcome measure of pain, which comprised a combination of the frequency and intensity of complaints of a certain severity, on the individual level. Thus, less severe conditions were not included as an outcome. The fact that the study population included 1115 women in common professions at a total of 116 different workplaces in Sweden is also a strength, as it increases the generalisability of the study.

For most of the measures, the participants at follow-up and the drop-outs reported similar frequencies of pain at baseline, and also had the same values of the sum score of ergonomic factors. The drop-outs tended to be more affected by neck pain and they reported a somewhat higher sum score for psychosocial factors. Furthermore, the partial drop-outs had somewhat lower frequencies of ≥ four pain sites at baseline. These differences were minor and we do not believe that they have influenced the results to any major extent.

An obvious limitation of this study is that it was limited to women, and the results may not be applicable to men. Further limitations of this longitudinal study are that all the data were self-reported, and were only compared on two occasions separated by a relatively long time (28 months, on average). It was therefore not possible to identify any changes that may have occurred between the two measurements.

Another limitation is that the data were collected over an extended period of time, at both baseline and follow-up. This was due to the administration required for the large number of different workplaces, and the fact that time-consuming assessments were performed (by objective measurements) of the physical workload and clinical examinations for certain subgroups at baseline [13]. However, the questionnaires were sent out to subsets that altered between the nursing staff, the sonographers and the teachers at both baseline and at follow-up. Thus, any societal changes that may have occurred during the period of the study would have affected the occupational groups similarly. Consequently, the mean follow-up period was similar in the five occupational groups, although there was considerable variation between the workplaces and between the participants. This variation in follow-up period may have influenced the results. However, the correlation between the length of the follow-up periods and change in number of pain sites was very low (rho 0.03) and we therefore believe that this was not a major problem.

Although the dimension job demands in the JCQ is intended to assess psychological demands at work, there may have been some overlap as some of the questions (especially those regarding hard work and high working pace) also could involve physical demands. For example, a high working pace often involves more stress, but may also lead to more lifting, faster movements, more frequent strenuous moments or more intense work in constrained postures. We do not think it is possible to completely distinguish between these exposures. However, as the psychosocial conditions in the workplace were asked for in the questionnaire, we still believe that the questions mainly are interpreted and responded to as psychological demands, and therefore of value of the study.

The purpose of sum scores was to reduce the uncertainty that may arise in analyses of separate dimensions including only a few questions, which was the case in some of the psychosocial dimensions. Then, sum scores give more robust estimates of associations. To be consistent, we also chose to create a sum score for ergonomic factors. An additional concern was that different dimensions of work-exposure were correlated, which makes it harder to single out the importance of each of them. Thus, the sum scores were created with the intention of investigating a gradually increasing exposure to several ergonomic and psychosocial factors. The underlying assumption was that experiencing two or more adverse ergonomic or psychosocial dimensions is worse than experiencing none or only one such dimension.

### The results in relation to other studies

A clear finding of this study was that pain at baseline was the strongest risk factor for pain at follow-up. Similar results, that previous pain episodes are predictors of present pain, have been reported previously [39, 40]. However, pain was associated with several occupational factors already at baseline [13]. Since many of the participants had worked for a long time (mean seniority 17 years), they may have developed work-related pain prior to the baseline study, which remained (or recurred) at follow-up. Persistent or recurrent pain may also be due to individual susceptibility or other non-work-related factors. Thus, to explore possible associations between occupational and personal risk factors, and changes in pain during the follow-up period, we adjusted for pain at baseline, and found longitudinal effects of adverse ergonomic conditions and psychosocial factors.

A general finding was that ergonomic and psychosocial factors were risk factors for both a high number of pain sites and specific sites of pain. These findings are consistent with the findings reported by Herin et al. in 2014 [41], but do not support the suggestion of Coggon et al. [27] that associations with risk factors differed importantly between a high number of pain sites and pain involving a small number of sites. Furthermore, our results indicate an incremental increase in the associations; the more adverse ergonomic and psychosocial working conditions reported at baseline, the higher the number of pain sites at follow-up (as illustrated in Figs. 2 and 3).

Among the personal and life-style factors, only a few were found to be associated with a high number of pain sites or specific sites of pain at follow-up. The prevalence of musculoskeletal pain is commonly believed to increase with progressing age [1, 27], but in the present study, this was only observed for pain in the hands and feet. High age did not explain a high number of pain sites. A high BMI was found to be of importance for pain in the feet, and for a high number of pain sites. However, it is likely that these associations were already present at baseline, since the statistical significances disappeared after adjustment for pain at baseline. Lack of time for personal relaxation was also found to be a risk factor for pain in the neck and shoulders. Unexpectedly, much domestic work at baseline was found to be a protective factor for pain in the hands and the feet at follow-up. We have no explanation of this, but it could be speculated that participants with persistent pain in the hands or feet need more rest after work, and thus avoid domestic work. Moreover, since hand and foot pain were associated with increasing age, another possible explanation could be that older participants no longer had children living at home, and thus had less need for domestic work. Daily smoking is considered to be a risk factor for poor health, including musculoskeletal pain [18]. However, we found that daily smoking was a protective factor for neck pain. Smokers may take more frequent breaks at work, which may be beneficial in relation to work-related pain. However, very few participants (4%) were daily smokers, and it cannot be ruled out that the protective effect was as a random finding.

There was considerable fluctuation in number of sites of pain between baseline and follow-up. About one third reported pain at an increased number of sites at follow-up, indicating a worsened condition. Fortunately, almost as many reported fewer pain sites at follow-up, and thus improved health in this regard. The occurrence of pain therefore appears not to be a static condition in many individuals, but rather reflects a pattern of recurrence [24, 25]. Nevertheless, in spite of the strict criterion for the outcome of pain, multiple pain sites were common in the present study population. Almost one fifth (18%) reported persistent or recurrent ≥ four pain sites at both baseline and follow-up. It is notable that only 11% reported no pain at any anatomical site, either at baseline or at follow-up. To the best of our knowledge, our definition of pain has not previously been used as an outcome measure in scientific studies of multisite pain, thus making direct comparisons difficult. However, according to a longitudinal study of Finnish food company workers, 36% of the study population (involving an unclear distribution of women and men) had “no pain” [25]. The high numbers of affected workers in the present study is worrying, since the study population was engaged in occupations which constitute a large proportion of the female labour force in Sweden. We have no reason to believe that the participants in this study were particularly burdened, as we studied employees at many different workplaces. Rather, factors associated with the organisational climate [42], such as time pressure and high physical/mental demands, may contribute to the disorders, as such conditions affect both the physical and psychosocial working conditions.

Thus, improvements in the working conditions for these occupations are required. Since both physical and psychosocial factors were of importance for musculoskeletal pain, neither area should be overlooked when implementing preventive measures. The occupational categories making up this cohort had different occupational exposures, which have been reported in the baseline-study [13]. Thus, different preventive actions should be prioritized in the different groups: The surgical nurses reported the highest scores regarding strenuous work posture and movements at baseline of the present study. The sonographers included in this study have been the subject of a more detailed study in which awkward postures (due to inappropriate workstation arrangement) and poor visual conditions were identified as the main risk factors for pain [15]. These factors were all included in the mechanical exposure index. Tailored ergonomic interventions are thus required for these groups, in order to alleviate physical strain. The assistant nurses reported the highest scores in physical activity and lifting at baseline [13] and the teachers reported the highest scores in most of the psychosocial dimensions [13]. The teachers may benefit from actions that reduce the demands placed on them, for example, improved organisation and fewer administrative tasks [16]. Measures to reduce the time pressure may be beneficial in all the occupational categories investigated in this study.

## Conclusions

The findings of the present study contributes to the knowledge that an overwhelming majority of the women in these common female-dominated occupations were affected by musculoskeletal pain, in one or several regions of the body. Furthermore, a substantial proportion of the women were classified as having persistent (or recurrent) ≥ four pain sites at both occasions. Since the present criterion for pain was rather strict, the reported impaired health conditions should not be overlooked. Among the occupational factors investigated, both ergonomic and psychosocial factors were found to be of importance for specific pain sites, as well as for a high number of pain sites. These findings are important in identifying suitable preventive measures in the working environment. Such preventive actions are needed on the individual, organizational and societal level.

## Supplementary information

**Additional file 1: Table S1.** Results from single-exposure models regarding the separate dimensions included in the sum scores of ergonomic and psychosocial factors.

**Additional file 2: Table S2.** Results from multi-exposure models regarding the separate dimensions included in the sum scores of ergonomic and psychosocial factors.

**Additional file 3: Table S3.** Single-exposure models of risk factors for specific pain sites.

## Data Availability

As the database used in the study described in this paper contains direct or indirect identifiers, it is not available in an open-access repository, as there is a possibility that the participants may not be completely anonymous. Readers interested in exploring the data should contact the corresponding author.

## Abbreviations

BMI: Body Mass Index
MEI: Mechanical exposure index
PHYI: Physical exposure index
JCQ: Job Content Questionnaire
