# Supplementary material for: The impact of occupational and personal factors on musculoskeletal pain - a cohort study of female nurses, sonographers and teachers
Source: BMC Musculoskelet Disord. 2020 Sep 18;21:621. doi: 10.1186/s12891-020-03640-4 (PMC7501652; doi:10.1186/s12891-020-03640-4)
Supplement: Supplementary file 1 — Additional file 1: Table S1. Results from single-exposure models regarding the separate dimensions included in the sum scores of ergonomic and psychosocial factors. [file 12891_2020_3640_MOESM1_ESM.docx]

**Additional table 1.** Single exposure models between the dimensions included in the sum-scores of ergonomic and psychosocial factors (at baseline), and musculoskeletal *pain* (at follow-up). The number of pain sites (stratified into five categories: 0, 1, 2, 3 and ≥ 4 sites) analysed using ordinal regression with odds ratios (ORs) and 95% confidence intervals (CIs). The outcomes of the specific anatomical regions (the neck, shoulders, hands, low back and feet) were estimated using Poisson regression as prevalence ratio (PR) and 95% confidence intervals (CIs). Results in bold face are statistically significant.

|  |  | Multisite pain |  | Specific pain sites | | | | | | | | |
| --- | --- | --- | --- | --- | --- | --- | --- | --- | --- | --- | --- | --- |
|  |  | Number of pain sites^a^  (N=1059) |  | Neck  (N = 1099) |  | Shoulders  (N= 1090) |  | Hands  (N = 1106) |  | Lower back  (N =1092) |  | Feet  (N =1105) |
|  | *N* | OR (CI) |  | PR (CI) |  | PR (CI) |  | PR (CI) |  | PR (CI) |  | PR (CI) |
|  |  |  |  |  |  |  |  |  |  |  |  |  |
| **Ergonomic factors** |  |  |  |  |  |  |  |  |  |  |  |  |
| Mechanical Exposure Index | 1056 |  |  |  |  |  |  |  |  |  |  |  |
| No exposure | 56 | 1 |  | 1 |  | 1 |  | 1 |  | **1** |  | 1 |
| Low | 160 | 1.61 (0.89 - 2.92) |  | 1.23 (0.72 – 2.10) |  | 1.76 (0.96 – 3.23) |  | 0.70 (0.37 – 1.36) |  | 1.30 (0.75 – 2.27) |  | 0.97 (0.46 – 2.05) |
| Medium | 393 | **3.28 (1.89 - 5.70)** |  | **1.72 (1.05 – 2.81)** |  | **2.38 (1.34 – 4.22)** |  | 1.39 (0.80 – 2.41) |  | **1.81 (1.08 – 3.03)** |  | 1.23 (0.62 – 2.41) |
| High | 447 | **4.80 (2.77 - 8.32)** |  | **2.17 (1.33 – 3.52)** |  | **3.02 (1.71 – 5.33)** |  | 1.59 (0.92 – 2.74) |  | **2.00 (1.20 – 3.33)** |  | 1.37 (0.70 – 2.68) |
|  |  |  |  |  |  |  |  |  |  |  |  |  |
| Physical exposure index | 1063 |  |  |  |  |  |  |  |  |  |  |  |
| No exposure | 113 | **1** |  | 1 |  | 1 |  | 1 |  | 1 |  | 1 |
| Low | 288 | 0.93 (0.62 - 1.39) |  | 1.00 (0.77 – 1.31) |  | 1.07 (0.84 – 1.37) |  | 0.90 (0.60 – 1.35) |  | 0.94 (0.71 – 1.25) |  | 0.72 (0.42 – 1.23) |
| Medium | 353 | 1.06 (0.72 – 1.56) |  | 0.93 (0.72 – 1.21) |  | 0.93 (0.73 – 1.19) |  | 1.09 (0.74 – 1.59) |  | 1.00 (0.76 – 1.31) |  | 1.19 (0.74 – 1.92) |
| High | 309 | **1.62 (1.09 – 2.40)** |  | 1.18 (0.92 – 1.52) |  | 1.14 (0.90 – 1.45) |  | **1.54 (1.07 – 2.23)** |  | 1.14 (0.87 – 1.48) |  | 1.48 (0.92 – 2.36) |
|  |  |  |  |  |  |  |  |  |  |  |  |  |
| Sensory demands | 1088 |  |  |  |  |  |  |  |  |  |  |  |
| Lowest quartile | 266 | 1 |  | 1 |  | 1 |  | 1 |  | 1 |  | 1 |
| 2 | 216 | 1.16 (0.83-1.61) |  | 0.94 (0.75 – 1.19) |  | 1.07 (0.86 – 1.33) |  | 1.20 (0.86 – 1.69) |  | 1.13 (0.89 – 1.42) |  | 1.10 (0.75 – 1.61) |
| 3 | 341 | **1.35 (1.00 – 1.81)** |  | 1.08 (0.89 – 1.32) |  | 1.15 (0.95 – 1.39) |  | **1.46 (1.09 – 1.96)** |  | 1.14 (0.93 – 1.41) |  | 0.92 (0.64 – 1.32) |
| Highest quartile | 265 | **1.89 (1.38 – 2.58)** |  | **1.30 (1.07 – 1.58)** |  | **1.32 (1.09 – 1.59)** |  | **1.68 (1.25 – 2.26)** |  | **1.24 (1.00 – 1.54)** |  | 1.09 (0.76 – 1.56) |
|  |  |  |  |  |  |  |  |  |  |  |  |  |
| **Psychosocial factors** |  |  |  |  |  |  |  |  |  |  |  |  |
| Job demands | 1097 |  |  |  |  |  |  |  |  |  |  |  |
| 1^st^ to 3^rd^ quartile | 820 | 1 |  | 1 |  | 1 |  | 1 |  | **1** |  | 1 |
| Highest quartile | 277 | **1.50 (1.17 – 1.93)** |  | **1.20 (1.03 – 1.39)** |  | **1.20 (1.04 – 1.38)** |  | 1.14 (0.91 – 1.41) |  | **1.26 (1.08 – 1.47)** |  | 1.11 (0.83 – 1.48) |
|  |  |  |  |  |  |  |  |  |  |  |  |  |
| Job control | 1097 |  |  |  |  |  |  |  |  |  |  |  |
| 2^nd^ to 4^th^ quartile | 810 | 1 |  | 1 |  | 1 |  | 1 |  | 1 |  | 1 |
| Lowest quartile | 287 | **1.52 (1.19 – 1.93)** |  | **1.26 (1.09 – 1.46)** |  | **1.17 (1.02 – 1.35)** |  | 1.19 (0.96 – 1.47) |  | **1.18 (1.01 – 1.37)** |  | 1.03 (0.77 – 1.37) |
|  |  |  |  |  |  |  |  |  |  |  |  |  |
| Job support from colleagues | 1093 |  |  |  |  |  |  |  |  |  |  |  |
| 2^nd^ to 4^th^ quartile | 860 | 1 |  | 1 |  | 1 |  | 1 |  | 1 |  | 1 |
| Lowest quartile | 233 | 1.29 (0.99 – 1.68) |  | **1.19 (1.02 – 1.39)** |  | **1.16 (1.00 – 1.35)** |  | 1.18 (0.93 – 1.48) |  | 1.08 (0.91 – 1.29) |  | 1.05 (0.77 – 1.42) |
|  |  |  |  |  |  |  |  |  |  |  |  |  |
| Emotional demands | 1088 |  |  |  |  |  |  |  |  |  |  |  |
| 1^st^ to 3^rd^ quartile | 878 | 1 |  | **1** |  | **1** |  | 1 |  | 1 |  | 1 |
| Highest quartile | 210 | **1.33 (1.00 – 1.77)** |  | 1.17 (0.99 – 1.38) |  | 1.06 (0.90 – 1.24) |  | 1.08 (0.84 – 1.37) |  | 1.08 (0.90 – 1.29) |  | 1.14 /0.84 – 1.56) |
|  |  |  |  |  |  |  |  |  |  |  |  |  |
| Demands of hiding emotions | 1087 |  |  |  |  |  |  |  |  |  |  |  |
| 1^st^ to 3^rd^ quartile | 902 | 1 |  | 1 |  | **1** |  | 1 |  | 1 |  |  |
| Highest quartile | 185 | **1.69 (1.27 – 2.25)** |  | **1.21 (1.02 – 1.43)** |  | **1.28 (1.10 – 1.49)** |  | **1.44 (1.15 – 1.81)** |  | **1.22 (1.03 – 1.45)** |  | 0.74 (0.50 – 1.09) |
|  |  |  |  |  |  |  |  |  |  |  |  |  |
| Leadership | 1088 |  |  |  |  |  |  |  |  |  |  |  |
| 2^nd^ to 4^th^ quartile | 852 | 1 |  | 1 |  | 1 |  | **1** |  | 1 |  | 1 |
| Lowest quartile | 234 | 1.19 (0.92 – 1.54) |  | 1.11 (0.94 – 1.30) |  | 1.11 (0.95 – 1.29) |  | 0.87 (0.67 – 1.12) |  | 1.08 (0.91 – 1.29) |  | 1.03 (0.76 – 1.41) |
|  |  |  |  |  |  |  |  |  |  |  |  |  |

^a^ Number of pain sites stratified into five categories: 0, 1, 2, 3 and ≥ 4 sites),
