# Supplementary material for: The impact of occupational and personal factors on musculoskeletal pain - a cohort study of female nurses, sonographers and teachers
Source: BMC Musculoskelet Disord. 2020 Sep 18;21:621. doi: 10.1186/s12891-020-03640-4 (PMC7501652; doi:10.1186/s12891-020-03640-4)
Supplement: Supplementary file 2 — Additional file 2: Table S2. Results from multi-exposure models regarding the separate dimensions included in the sum scores of ergonomic and psychosocial factors. [file 12891_2020_3640_MOESM2_ESM.docx]

**Additional table 2.** Multi exposure models^a^ between the variables included in the sum-scores of ergonomic and psychosocial factors (at baseline), and musculoskeletal *pain* (at follow-up). The number of pain sites (stratified into five categories: 0, 1, 2, 3 and ≥ 4 sites) was analysed using ordinal regression with odds ratios (ORs) and 95% confidence intervals (CIs). The outcomes of the specific anatomical regions (the neck, shoulders, hands, low back and feet) were estimated using Poisson regression as prevalence ratio (PR) and 95% confidence intervals (CIs). All models were adjusted for personal- and life style factors ^a^. Results in bold face are statistically significant.

|  |  | Multisite pain |  | Specific pain sites | | | | | | | | |
| --- | --- | --- | --- | --- | --- | --- | --- | --- | --- | --- | --- | --- |
|  |  | Number of pain sites^a^  (N=1059) |  | Neck  (N = 1099) |  | Shoulders  (N= 1090) |  | Hands  (N = 1106) |  | Lower back  (N =1092) |  | Feet  (N =1105) |
|  | *N* | OR (CI) |  | PR (CI) |  | PR (CI) |  | PR (CI) |  | PR (CI) |  | PR (CI) |
|  |  |  |  |  |  |  |  |  |  |  |  |  |
| **Ergonomic factors** |  |  |  |  |  |  |  |  |  |  |  |  |
| Mechanical Exposure Index | 1056 |  |  |  |  |  |  |  |  |  |  |  |
| No exposure | 56 | 1 |  | 1 |  | 1 |  | 1 |  | 1 |  | 1 |
| Low | 160 | 1.59 (0.82 – 3.09) |  | 1.25 (0.71 - 2.20) |  | 1.50 (0.80 - 2.80) |  | 0.57 (0.28 - 1.17) |  | 1.37 (0.77 - 2.44) |  | 1.19 (0.50 - 2.82) |
| Medium | 393 | **2.92 (1.52 – 5.62)** |  | **1.77 (1.03 - 3.04)** |  | **2.11 (1.16 - 3.85)** |  | 0.96 (0.50 - 1.83) |  | **1.86 (1.07 - 3.21)** |  | 1.40 (0.61 - 3.23) |
| High | 447 | **3.63 (1.80 – 7.32)** |  | **2.15 (1.22 - 3.77)** |  | **2.71 (1.47 - 5.00)** |  | 0.93 (0.47 - 1.87) |  | **1.90 (1.07 - 3.36)** |  | 1.58 (0.65 - 3.82) |
|  |  |  |  |  |  |  |  |  |  |  |  |  |
| Physical exposure index | 1063 |  |  |  |  |  |  |  |  |  |  |  |
| No exposure | 113 | 1 |  | 1 |  | 1 |  | 1 |  | 1 |  | 1 |
| Low | 288 | 0.88 (0.57 – 1.35) |  | 0.97 (0.75 - 1.25) |  | 1.04 (0.83 - 1.31) |  | 0.97 (0.64 - 1.49) |  | 0.92 (0.69 - 1.23) |  | 0.73 (0.41 - 1.30) |
| Medium | 353 | 0.86 (0.54 – 1.37) |  | 0.89 (0.67 - 1.18) |  | 0.86 (0.66 - 1.11) |  | 1.13 (0.71 - 1.78) |  | 0.90 (0.66 - 1.23) |  | 0.89 (0.49 - 1.64) |
| High | 309 | 1.17 (0.69 – 2.00) |  | 1.06 (0.78 - 1.44) |  | 0.95 (0.71 - 1.27) |  | 1.44 (0.87 - 2.37) |  | 0.92 (0.65 - 1.30) |  | 1.12 (0.58 - 2.17) |
|  |  |  |  |  |  |  |  |  |  |  |  |  |
| Sensory demands | 1088 |  |  |  |  |  |  |  |  |  |  |  |
| Lowest quartile | 266 | 1 |  | 1 |  | 1 |  | 1 |  | 1 |  | 1 |
| 2 | 216 | 0.83 (0.57-1.21) |  | 0.80 (0.63 - 1.02) |  | 0.91 (0.73 - 1.13) |  | 0.94 (0.65 - 1.36) |  | 1.04 (0.81 - 1.33) |  | 1.12 (0.75 - 1.68) |
| 3 | 341 | 0.84 (0.58-1.21) |  | 0.82 (0.66 - 1.03) |  | 0.87 (0.71 - 1.07) |  | 1.09 (0.77 - 1.55) |  | 0.96 (0.75 - 1.23) |  | 1.01 (0.66 - 1.55) |
| Highest quartile | 265 | 0.94 (0.62-1.42) |  | 0.90 (0.70 - 1.15) |  | 0.84 (0.67 - 1.06) |  | 1.09 (0.74 - 1.60) |  | 0.98 (0.74 - 1.29) |  | 1.18 (0.74 - 1.86) |
|  |  |  |  |  |  |  |  |  |  |  |  |  |
| **Psychosocial factors** |  |  |  |  |  |  |  |  |  |  |  |  |
| Job demands | 1097 |  |  |  |  |  |  |  |  |  |  |  |
| 1^st^ to 3^rd^ quartile | 820 | 1 |  | 1 |  | 1 |  | 1 |  | 1 |  | 1 |
| Highest quartile | 277 | 1.29 (0.93 - 1.77) |  | 1.12 (0.94 - 1.34) |  | 1.14 (0.96 - 1.34) |  | 1.13 (0.87 - 1.48) |  | **1.21 (1.00 - 1.47)** |  | 1.00 (0.71 - 1.40) |
|  |  |  |  |  |  |  |  |  |  |  |  |  |
| Job control | 1097 |  |  |  |  |  |  |  |  |  |  |  |
| 2^nd^ to 4^th^ quartile | 810 | 1 |  | 1 |  | 1 |  | 1 |  | 1 |  | 1 |
| Lowest quartile | 287 | 1.28 (0.96 - 1.71) |  | **1.21 (1.02 - 1.43)** |  | 1.08 (0.92 - 1.26) |  | 0.98 (0.77 - 1.24) |  | 1.10 (0.92 - 1.31) |  | 0.99 (0.71 - 1.38) |
|  |  |  |  |  |  |  |  |  |  |  |  |  |
| Job support from colleagues | 1093 |  |  |  |  |  |  |  |  |  |  |  |
| 2^nd^ to 4^th^ quartile | 860 | 1 |  | 1 |  | 1 |  | 1 |  | 1 |  | 1 |
| Lowest quartile | 233 | 1.19 (0.89 - 1.61) |  | 1.17 (1.00 - 1.38) |  | 1.13 (0.97 - 1.32) |  | 1.19 (0.94 - 1.52) |  | 1.00 (0.83 - 1.20) |  | 1.03 (0.74 - 1.44) |
|  |  |  |  |  |  |  |  |  |  |  |  |  |
| Emotional demands | 1088 |  |  |  |  |  |  |  |  |  |  |  |
| 1^st^ to 3^rd^ quartile | 878 | **1** |  | 1 |  | 1 |  | 1 |  | 1 |  | 1 |
| Highest quartile | 210 | **1.76 (1.19 - 2.59)** |  | 1.22 (0.98 - 1.51) |  | 1.22 (0.99 - 1.50) |  | 1.15 (0.84 - 1.58) |  | 1.13 (0.90 - 1.42) |  | 1.25 (0.85 - 1.85) |
|  |  |  |  |  |  |  |  |  |  |  |  |  |
| Demands of hiding emotions | 1087 |  |  |  |  |  |  |  |  |  |  |  |
| 1^st^ to 3^rd^ quartile | 902 | **1** |  | 1 |  | 1 |  | 1 |  | 1 |  | 1 |
| Highest quartile | 185 | **1.42 (1.02 - 1.98)** |  | 1.04 (0.87 - 1.24) |  | 1.12 (0.95 - 1.31) |  | **1.38 (1.07 - 1.78)** |  | 1.12 (0.92 - 1.36) |  | 0.68 (0.45 - 1.03) |
|  |  |  |  |  |  |  |  |  |  |  |  |  |
| Leadership | 1088 |  |  |  |  |  |  |  |  |  |  |  |
| 2^nd^ to 4^th^ quartile | 852 | 1 |  | 1 |  | 1 |  | 1 |  | 1 |  | 1 |
| Lowest quartile | 234 | 1.07 (0.79 - 1.45) |  | 1.00 (0.84 - 1.19) |  | 1.01 (0.87 - 1.18) |  | 0.85 (0.65 - 1.11) |  | 1.00 (0.83 - 1.21) |  | 1.05 (0.76 - 1.46) |
|  |  |  |  |  |  |  |  |  |  |  |  |  |

^a^ All models include mechanical exposure index, physical exposure index, sensory demands, job demands, job control, job support from colleagues, emotional demands, demands of hiding emotions, leadership, computer work, age, BMI, personal relaxation, domestic work, physical exercise, smoking and occupational category.
