# Supplementary material for: The impact of occupational and personal factors on musculoskeletal pain - a cohort study of female nurses, sonographers and teachers
Source: BMC Musculoskelet Disord. 2020 Sep 18;21:621. doi: 10.1186/s12891-020-03640-4 (PMC7501652; doi:10.1186/s12891-020-03640-4)
Supplement: Supplementary file 3 — Additional file 3: Table S3. Single-exposure models of risk factors for specific pain sites. [file 12891_2020_3640_MOESM3_ESM.docx]

**Additional Table 3.** Single-exposure models in the total study population (n=1115) of associations between self-reported ergonomic, psychosocial and personal factors at baseline, and musculoskeletal pain in the neck, shoulders, hands, lower back and feet at follow-up, calculated with Poisson regression, with overall *p*-values, prevalence ratios (PRs) and 95% confidence intervals (CIs)

|  |  | Neck (N=1099) | | Shoulders (N=1090) | | Hands (N=1106) | | Lower back (N=1092) | | Feet (N=1105) | |
| --- | --- | --- | --- | --- | --- | --- | --- | --- | --- | --- | --- |
|  | N | *p* | PR (CI 95%) | *p* | PR (CI 95%) | *p* | PR (CI 95%) | *p* | PR (CI 95%) | *p* | PR (CI 95%) |
|  |  |  |  |  |  |  |  |  |  |  |  |
| Pain at baseline^a^ |  | **<0.001** |  | **<0.001** |  | **<0.001** |  | **<0.001** |  | **<0.001** |  |
| *no* |  |  | 1 |  | 1 |  | **1** |  | **1** |  | **1** |
| *yes* |  |  | **3.37 (2.88-3.93)** |  | **2.68 (2.31-3.09)** |  | **4.04 (3.35-4.87)** |  | **2.93 (2.51-3.41)** |  | **4.66 (3.70-5.89)** |
|  |  |  |  |  |  |  |  |  |  |  |  |
| Sum score of ergonomic factors (*scale*)^b^ | 1046 | **<0.001** |  | **<0.001** |  | **<0.001** |  | **0.004** |  | **0.02** |  |
| *1* | 211 |  | 1 |  | 1 |  | 1 |  | 1 |  | 1 |
| *2* | 283 |  | 1.17 (0.92-1.50) |  | 1.07 (0.85-1.35) |  | 1.20 (0.82-1.75) |  | 1.24 (0.96-1,60) |  | 1.06 (0.69-1.62) |
| *3* | 321 |  | **1.33 (1.06-1.68)** |  | **1.36 (1.10-1.67)** |  | **1.95 (1.39-2.72)** |  | **1.49 (1.17-1.89)** |  | 1.20 (0.81-1.80) |
| *4* | 231 |  | **1.58 (1.26-2.0)** |  | **1.41 (1.14 – 1.76)** |  | **1.89 (1.33-2.69)** |  | **1.36 (1.05-1.76)** |  | **1.56 (1.04-2.33)** |
|  |  |  |  |  |  |  |  |  |  |  |  |
| Complaints re: computer workstation arrangements^b^ | 1065 | **<0.01** |  | 0.06 |  | 0.19 |  | 0.27 |  | 0.64 |  |
| *Satisfied* | 463 |  | 1 |  | 1 |  | 1 |  | 1 |  | 1 |
| *Neutral* | 341 |  | 1.18 (0.99-1.39) |  | 1.03 (0.88-1.21) |  | 0.93 (0.74-1.17) |  | **1.24 (1.05-1.47)** |  | 1.21 (0.91-1.62) |
| *Dissatisfied* | 261 |  | **1.29 (1.08-1.54)** |  | **1.18 (1.00-1.38)** |  | 0.84 (0.65-1.09) |  | 1.07 (0.88-1.31) |  | 0.87 (0.61-1.24) |
|  |  |  |  |  |  |  |  |  |  |  |  |
| Sum score of psychosocial factors (*scale*) ^b^ | 1070 | **<0.001** |  | **<0.001** |  | **0.03** |  | **0.001** |  | 0.86 |  |
| *0* | 337 |  | 1 |  | 1 |  | 1 |  | 1 |  | 1 |
| *1* | 339 |  | 1.23 (1.01-1.49) |  | 1.04 (0.87-1.25) |  | **1.48 (1.14-1.93)** |  | 1.16(0.96-1.42) |  | 1.31 (0.95-1.81) |
| *2* | 203 |  | 1.28 (1.04-1.59) |  | 1.16 (0.96-1.42) |  | 1.02 (0.72-1.42) |  | 1.22 (0.98-1.53) |  | 0.95 (0.63-1.43) |
| *3* | 130 |  | 1.51 (1.21-1.89) |  | **1.34 (1.09-1.65)** |  | **1.65 (1.20-2.27)** |  | **1.37(1.08-1.74)** |  | 1.34 (0.89-2.04) |
| *4* | *45* |  | 1.74 (1.32-2.31) |  | **1.53 (1.18-2.00)** |  | 1.27 (0.75-2.16) |  | **1.55 (1.14-2.12)** |  | 0.56 (0.21-1.48) |
| *5* | *16* |  | 1.63 (1.03-2.58) |  | **1.70 (1.19-2.42)** |  | **1.91 (0.99-3.67)** |  | 1.46 (0.87-2.43) |  | 1.58 (0.65-3.82) |
|  |  |  |  |  |  |  |  |  |  |  |  |
| Age group (*years*)^c^ | 1115 | 0.38 |  | 0.07 |  | **0.001** |  | 0.44 |  | **<0.001** |  |
| *<40* | 267 |  | 1 |  | 1 |  | 1 |  | 1 |  | 1 |
| *40-55* | 544 |  | 0.93 (0.79-1.10) |  | 0.91 (0.78-1.06) |  | 1.22 (0.93-1.62) |  | 1.13 (0.93-1.36) |  | **2.49 (1.61-3.84)** |
| *>55* | 304 |  | 0.87 (0.72-1.06) |  | **0.81 (0.67-0.97)** |  | **1.63 (1.23-2.17)** |  | 1.06 (0.86-1.31) |  | **2.50 (1.58-3.95)** |
|  |  |  |  |  |  |  |  |  |  |  |  |
| Body Mass Index (*points*)^c^ | 1092 | 0.93 |  | 0.64 |  | 0.08 |  | 0.08 |  | **<0.001** |  |
| *<18.5* | 11 |  | 1.09 (0.57-2.09) |  | 1.02 (0.53-1.97) |  | 1.11 (0.42-2.93) |  | 0.71 (0.27-1.87) |  | 0.59 (0.09-3.85) |
| *18.5-24.9* | 707 |  | 1 |  | 1 |  | 1 |  | 1 |  | 1 |
| *25.0-29.9* | 287 |  | 1.04 (0.89-1.22) |  | 1.09 (0.94-1.26) |  | 1.14 (0.91-1.43) |  | 1.11 (0.94-1.31) |  | 1.19 (0.88-1.61) |
| *>30* | 87 |  | 1.06 (0.82-1.36) |  | 1.11 (0.88-1.40) |  | **1.49 (1.10-2.02)** |  | **1.31 (1.04-1.65)** |  | **2.16 (1.53-3.05)** |
|  |  |  |  |  |  |  |  |  |  |  |  |
| Personal recovery time^b^ | 1094 | **<0.001** |  | **<0.001** |  | 0.46 |  | 0.64 |  | 0.14 |  |
| *≥ 3 h /day* | 281 |  | 1 |  | 1 |  | 1 |  | 1 |  | 1 |
| *1-2 h/day* | 577 |  | 1.24 (1.03-1.50) |  | **1.26 (1.05-1.50)** |  | 0.92 (0.73-1.16) |  | 1.05 (0.88-1.25) |  | 1.02 (0.76-1.38) |
| *< 1 h/day* | 236 |  | **1.51 (1.24-1.86)** |  | **1.49 (1.23-1.81)** |  | 0.90 (0.68-1.20) |  | 1.05 (0.85-1.30) |  | 0.73 (0.49-1.10) |
|  |  |  |  |  |  |  |  |  |  |  |  |
| Domestic work^b^ | 1102 | 0.34 |  | 0.14 |  | **0.04** |  | 0.70 |  | 0.08 |  |
| *0-10 h/week* | 382 |  | 1 |  | 1 |  | 1 |  | 1 |  | 1 |
| *11-20 h/week* | 459 |  | 0.93 (0.79-1.10) |  | 0.94 (0.80-1.10) |  | 0.77 (0.62-0.97) |  | 1.02 (0.86-1.21) |  | 0.85 (0.64-1.13) |
| *≥ 21 h/week* | 261 |  | 1.11 (0.93-1.32**)** |  | 1.15 (0.98-1.36) |  | 0.78 (0.60-1.02) |  | 1.04 (0.86-1.26) |  | 0.73 (0.51-1.05) |
|  |  |  |  |  |  |  |  |  |  |  |  |
| Physical exercise^b^ | 1107 | **0.01** |  | 0.38 |  | 0.67 |  | **0.04** |  | 0.85 |  |
| *Twice a week or more* | 807 |  | 1 |  | 1 |  | 1 |  | 1 |  | 1 |
| *Once a week* | 153 |  | 1.15 (0.95-1.39) |  | 1.10 (0.92-1.32) |  | 0.95 (0.71-1.28) |  | 1.18 (0.91-1.37) |  | 0.81 (0.53-1.22) |
| *Occasionally or never* | 147 |  | 1.24 (1.03-1.49) |  | 1.06 (0.88-1.28) |  | 0.95 (0.70-1.28) |  | 1.21 (0.99-1.47) |  | 1.12 (0.78-1.60) |
|  |  |  |  |  |  |  |  |  |  |  |  |
| Daily smokers | 1111 | 0.52 |  | 0.81 |  | 0.13 |  | 0.06 |  | 0.62 |  |
| *no* | 1063 |  | 1 |  | 1 |  | 1 |  | 1 |  | 1 |
| *yes* | 48 |  | 0.89 (0.61-1.29) |  | 0.96 (0.69-1.33) |  | 1.36 (0.92-2.02) |  | 1.32 (0.99-1.75) |  | 0.84 (0.42-1.68) |
|  |  |  |  |  |  |  |  |  |  |  |  |
| Occupational category ^c^ | 1115 | **<0.01** |  | **<0.001** |  | **<0.001** |  | **<0.01** |  | **<0.01** |  |
| *Teacher* | 246 |  | 1 |  | 1 |  | 1 |  | 1 |  | 1 |
| *Anaesthetic nurse* | 214 |  | 0.81 (0.63-1.04) |  | 1.09 (0.87-1.38) |  | 1.18 (0.81 -1.72) |  | 0.82 (0.63-1.06) |  | 0.96 (0.64-1.43) |
| *Surgical nurse* | 209 |  | 1.20 (0.97-1.48) |  | 1.24 (1.0-1.55) |  | **1.62 (1.15-2.30)** |  | **1.26(1.02-1.58)** |  | 1.07 (0.72-1.58) |
| *Assistant nurse* | 224 |  | 1.07 (0.86-1.33) |  | 1.2 (0.96-1.49) |  | **2.12 (1.54-2.93)** |  | **1.26 (1.01-1.56)** |  | 1.39 (0.97-1.98) |
| *Sonographer* | 222 |  | **1.25 (1.02-1.53)** |  | **1.55 (1.27-1.89)** |  | **1.65 (1.17-2.32)** |  | 1.06 (0.84-1.33) |  | **0.57 (0.35-0.92)** |
|  |  |  |  |  |  |  |  |  |  |  |  |

^a^ The frequency of musculoskeletal pain in the different anatomical sites is given in Table 1.

^b^ Overall *p*-values are given as continuous variable.

^c^ Overall *p*-values are given as categorical variable “test of model effect”.
